# Assessing Treatment Outcomes of Chondromyxoid Fibroma: A Case Series

**DOI:** 10.7759/cureus.101344

**Published:** 2026-01-12

**Authors:** Zachary Butler, Dylan Riley, Michael Murray, Samuel Alfonsi, Austin Yu, Steven Gitelis, Alan T Blank

**Affiliations:** 1 Orthopaedic Oncology, Rush University Medical Center, Chicago, USA

**Keywords:** chondromyxoid fibroma, conservative management, non-operative treatment, observation, recurrence, transformation

## Abstract

Background: Chondromyxoid fibroma (CMF) is a rare, benign, and locally aggressive bone tumor. It presents diagnostic challenges due to its radiologic similarity to other lesions and variable recurrence rates following surgical treatment. Current literature lacks standardized treatment guidelines, and nonoperative management strategies have not been previously described.

Methods: We conducted a retrospective review of patients diagnosed with CMF at our institution between 2010 and 2024. Eight patients met the inclusion criteria, with treatment modalities including en bloc resection (n=2), intralesional curettage with adjuvants (n=4), and conservative management with serial imaging (n=2). Demographic, clinical, and treatment-related data were collected, and outcomes were analyzed.

Results: The cohort consisted of three males and five females, with a mean age of 55.3 ± 11.8 years and an average follow-up of 41.7 ± 28.5 months (range: eight to 104 months). Lesions were located in the distal femur (n=3), pelvis (n=2), clavicle (n=1), great toe (n=1), and T3 vertebral body (n=1). All surgical patients remained free of tumor recurrence, regardless of treatment modality. Two patients (25%) treated with en bloc resection experienced surgical complications requiring revision arthroplasty. Conservative management with serial imaging was successful in two asymptomatic patients (25%), with no evidence of disease progression over eight and 104 months, respectively.

Conclusion: This case series demonstrates that en bloc resection, intralesional curettage with adjuvants, and conservative management with serial imaging are viable options for managing CMF, depending on patient-specific factors. Notably, to the best of our knowledge, this is the first series to document successful nonoperative management of CMF with serial imaging in carefully selected asymptomatic patients. Our results add to the limited literature on CMF and propose that serial imaging could be a potential management strategy in select patients with CMF.

## Introduction

Chondromyxoid fibroma (CMF) is a rare, benign, locally aggressive tumor that presents diagnostic and therapeutic challenges due to its similarity to more common bone lesions [1]. First reported by Jaffe and Lichtenstein in 1948, CMF is found more commonly in males in the second or third decade of life. It most commonly affects the long bones, particularly the proximal tibia and distal femur, but can occur in the craniofacial bones as well [2,3]. Although histologically stellate-shaped cells are the hallmark of CMF, the mix of chondroid, myxoid, and fibrous elements often mimics other more common bone pathologies such as aneurysmal bone cyst, unicameral bone cyst, giant cell tumor, chondroblastoma, chondrosarcoma, or fibrous dysplasia [4-7]. This broad differential diagnosis highlights the difficulty of making an accurate diagnosis of CMF.

Due to the rarity of CMF, large series are rare, and there is no standard agreed-upon treatment in the field. The current literature highlights surgery as the primary treatment modality, with options including intralesional curettage, intralesional curettage with bone graft or cement, or resection [1,8]. Studies have reported variable outcomes and recurrence rates depending on the treatment modality. Lersundi et al. reported a recurrence rate of 38% following curettage alone, compared to 0% when treated with resection [5]. Similarly, Baker et al. demonstrated a low recurrence rate following excisional biopsy (0%), while reporting one recurrence in a patient treated with curettage alone [9]. Despite these findings, a literature review from Wangsiricharoen et al. reports a local recurrence rate ranging from 13% to 26%, with a small risk of malignant transformation [10]. Sehayik et al. reported the risk of malignant transformation in a case of CMF treated by curettage alone that recurred in three years as chondrosarcoma requiring a hemipelvectomy [11]. Malignant transformation was also noted by Wu et al., who reported two cases of CMF transformation out of their review of 278 cases (<1.0%). The first case was malignant fibrous histiocytoma of the pubis five months after diagnosis of CMF, the other case was fibrosarcoma arising in the proximal tibia six years after treatment, which included radiation therapy for CMF [12].

Given CMF’s benign nature and low malignant transformation risk, observation with serial imaging may represent a viable alternative to surgery in carefully selected cases where surgical morbidity outweighs oncologic risk, though this approach has not been previously reported. In this case series, we aim to report our institution's experience with the management of patients with CMF over the past 14 years. While limited by a small cohort size, we seek to analyze treatment strategies and outcomes to contribute to future treatment recommendations for this rare lesion. Therefore, the objectives of this study were to (1) report our institution's 14-year experience with CMF management, (2) analyze treatment strategies and clinical outcomes across surgical and non-surgical modalities, and (3) evaluate the feasibility of conservative management with serial imaging in select patients.

## Materials and methods

Following Institutional Review Board approval by the Rush University Medical Center (approval no. 24061002), a musculoskeletal pathologist conducted a natural language search within our institution's electronic medical record to identify all cases of chondromyxoid fibroma in patients over the age of 18 from 2010 to 2024. A total of 12 patients were initially identified. Patients with incomplete records or less than six months of follow-up were excluded to ensure an adequate assessment of treatment outcomes and detection of potentially early recurrences. This resulted in a final cohort of eight patients. 

Data collection

Electronic medical records of these eight patients were reviewed to determine demographic data, clinical presentation, treatment details, and outcomes. Treatment modalities included en bloc resection with distal femoral replacement (n=2, 25.0%), intralesional excision with electrocautery (n=2, 25.0%), intralesional excision with electrocautery and bone allograft (n=1, 12.5%), intralesional excision with bone cement (n=1, 12.5%), and non-operative management with serial imaging (n=2, 25.0%). Clinical outcomes assessed included local recurrence, disease progression, surgical complications, and functional status at final follow-up.

Statistical analyses

Demographic and clinical data were analyzed using descriptive statistics. Continuous variables of interest were represented as the mean, standard deviation (SD), or median, and range. Recurrence time was calculated from the time of surgical intervention until the date of local relapse. Given the descriptive nature of this case series and small sample size, statistical comparison between treatment groups was not performed.

## Results

Eight individuals were diagnosed with CMF at our institution from 2010 to 2024 and were included in the study. To facilitate reference throughout this article, patients were assigned the following codes: M1, M2, and M3 for males and F1, F2, F3, F4, and F5 for females. Table 1 shows this sample’s demographics. The overall cohort of patients consisted of three (37.5%) males and five (62.5%) females. Five (62.5%) patients identified as White (M1, M2, F2, F3, F5), two (25.0%) patients identified as Black (M3, F4), and one (12.5%) patient identified as Other (F1). The average BMI of the study population was 30.2 ± 3.2. The average age at the time of diagnosis was 55.3 ± 11.8 years, and the mean follow-up period was 41.7 ± 28.5 months. Six (75.0%) patients were experiencing pain on presentation (M1, M2, F1, F2, F4, F5), one of whom also observed a visible mass (M2). One (12.5%) presented with a palpable mass but denied pain (M3), and one (12.5%) patient was diagnosed incidentally after undergoing an MRI for unrelated neurologic symptoms (F3). Three (37.5%) lesions were localized to the distal femur (F1, M2, M3), two (25.0%) localized to the pelvis, one in the acetabulum (M1) and one in the iliac crest (F2), one (12.5%) localized to the T3 vertebral body (F3), one (12.5%) localized to the clavicle (F4), and one (12.5%) localized to the great toe (F5).

**Table 1:** Demographic and clinical characteristics of patients with CMF CMF: Chondromyxoid fibroma

Demographic and clinical characteristics		n (%)
Sex	Male	3 (37.5)
	Female	5 (62.5)
Race	White	5 (62.5)
	Black	2 (25.0)
	Other	1 (12.5)
Presenting symptom	Pain	6 (75.0)
	Mass	1 (12.5)
	Incidental	1 (12.5)

**Figure 1:**
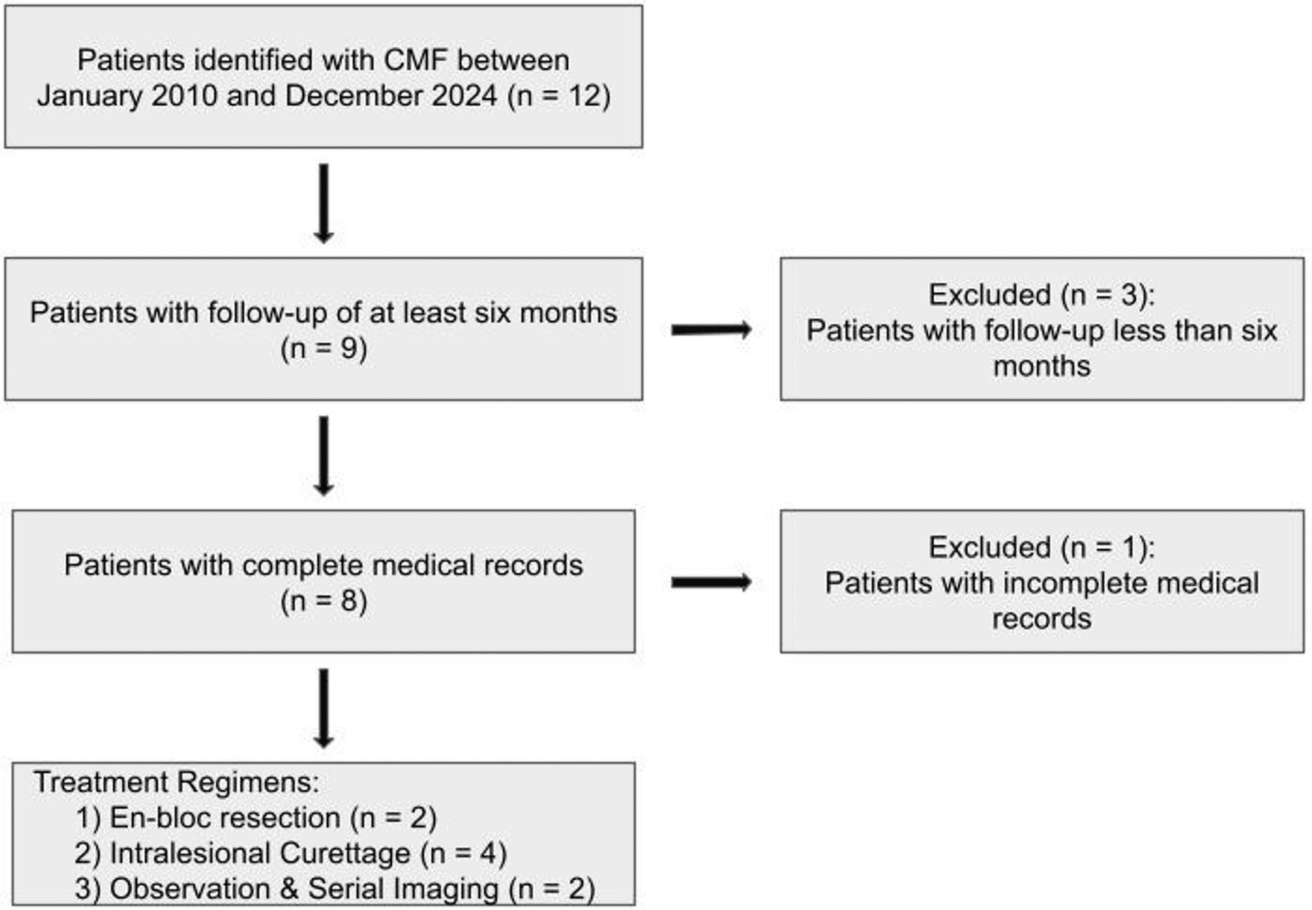
Patient selection and treatment allocation flow diagram CMF: Chondromyxoid fibroma

Six (75.0%) patients were treated surgically, two (25.0%) received en bloc resection, and four (50.0%) received intralesional curettage (Figure 1). The patients treated with en bloc resection (M3, F1), both of whom had distal femoral lesions, received modular oncology distal femoral replacements. Two patients were treated with intralesional excision plus electrocautery, and two patients were treated with curettage plus either cancellous bone allograft chips or injectable synthetic bone filler with plate fixation. At final follow-up, no patient demonstrated local recurrence or disease progression, regardless of treatment modality. The remaining two (25.0%) patients underwent core needle biopsy and were treated conservatively with serial imaging (F2, F3) due to their asymptomatic nature. One lesion was located in the T3 vertebral body, and one was in the iliac crest, with a follow-up period of 104.0 and 8.0 months, respectively. To date, there has been no evidence of progression (Table 2).

**Table 2:** Treatment modalities and outcomes for eight patients with CMF CMF: Chondromyxoid fibroma

Patient Code	Location	Treatment	Length of follow-up (months)	Recurrence/Progression
M1	Pelvis (acetabulum)	Intralesional curettage with bone allograft	49.6	No
M2	Femur (distal)	Intralesional curettage with bone cement and fixation	47.6	No
M3	Femur (distal)	En bloc resection	37.2	No
F1	Femur (distal)	En bloc resection	25.2	No
F2	Pelvis (iliac crest)	Observation + serial imaging	8.0	No
F3	T3 vertebral body	Observation + serial imaging	104.0	No
F4	Clavicle	Intralesional curettage with electrocautery	25.5	No
F5	Great toe	Intralesional curettage with electrocautery	36.7	No

Two cases presented with surgical complications following initial en bloc resection (M3, F1). One patient underwent revision after experiencing continued pain and gait difficulty. After several non-diagnostic studies, including joint aspiration, cultures, and a bone scan, it was decided that surgery was the best option to determine the pain source. Femoral loosening and severe patellar arthritis were found intraoperatively, leading to a modular oncology revision with patellectomy. The other patient underwent revision surgery after trauma resulted in tibial loosening 1.5 months after initial resection. Both revisions were performed for mechanical complications of the prosthesis rather than tumor recurrence, and both patients remain disease-free at final follow-up.

## Discussion

Chondromyxoid fibroma is a rare, slow-growing neoplasm most commonly found in the metaphysis of long bones that comprises only 0.5% of bony tumors [3]; its low incidence rate makes it a difficult neoplasm to analyze and establish consensus treatment. Additionally, because of the rarity of CMF and its challenging appearance to distinguish radiographically [3], early differential diagnoses often do not consider CMF as the primary diagnosis. Because most research on CMF is predominantly published as case series of patients treated over decades, there are few ultimate surgical treatment guidelines for CMF management, none of which have included observation with serial imaging [1].

In this case series, we aim to analyze the treatment options and outcomes of eight patients diagnosed with CMF at our institution over a 14-year period. Our series illustrates that en bloc resection, intralesional curettage with electrocautery, bone grafting or bone cement, and observation with serial radiographs are viable treatment modalities, with no patients experiencing tumor recurrence or progression at final follow-up. Two patients in our case series were treated with en bloc resection, which is noted to be the treatment modality that best avoids CMF recurrence [1]. The patients in our series who underwent en bloc resection had aggressive lesions encompassing the distal two-thirds of the femur. They both received modular oncology distal femoral replacements, which both eventually required revision surgery; however, neither of the two patients had CMF recurrence. In a case series of 41 patients with CMF, Lersundi et al. describe four patients with CMF lesions in the proximal tibia, pelvis, and proximal fibula, which were treated with resection; around the two-year follow-up, no patient had tumor recurrence [5], similar to our results. Similarly, in a case series of 31 patients, Karaca et al. found no CMF recurrence in five patients treated with resection [8]. With respect to resection, these findings coincide with the historical recommendation of wide excision or en bloc segmental resection of CMF, which traditionally has a recurrence rate as low as 4% [13]. Although resection is most successful in reducing CMF recurrence, it is best only for larger lesions or those in anatomic locations where resection procedures would not incur a higher risk of fracture or re-operation, limiting its indications. In our study, both CMF lesions treated with en bloc resection received modular oncology arthroplasty implantation.

Four patients in our case series were treated with intralesional curettage with an adjuvant, and none experienced recurrence of CMF. Two received electrocautery, and one received bone grafting, while the other received bone cement and plating. Historically, curettage alone often leads to a higher rate of recurrence; thus, adjuvants with either bone graft or bone cement are often used to reduce recurrence rates to those seen after resection [1,5,12,14]. In a series of 22 cases treated with intralesional curettage, four of which received adjuvant cementation, Bharma et al. describe a recurrence rate of only 9.1% [7]. Similarly, Karaca et al. found no recurrence in those patients treated with intralesional curettage with bone graft or bone cement in their case series [8]. These results, along with those seen in this case series, support intralesional curettage as an effective treatment option, especially for lesions with large intraosseous defects that need adjuvants to impart stability and decrease the risk of future fracture.

Most notably, two patients (25%) in our case series were successfully managed with observation and serial imaging alone, representing the first reported cases of non-operative CMF management. Neither patient experienced tumor progression. Given the absence of reported metastases from CMF and the low malignant transformation rate (1% to 2%), observation with serial imaging represents a rational approach in carefully selected patients. The patients in our study who underwent serial imaging had lesions in the iliac crest and the T3 vertebral body, both of which present high surgical complications and surgical morbidity. In select patients, such as this, serial radiographs and watchful waiting may be the appropriate management for lesions that are asymptomatic, small, and affect bones not directly subjected to weight-bearing forces. Our results demonstrate that serial radiographs may be the appropriate option for the asymptomatic patient who wants to avoid operative intervention and its potential complications, morbidity, need for rehabilitation, and risk for CMF recurrence, which is seen with intralesional curettage. In select cases with a high surgical morbidity, if these tumors were to progress, newer techniques have been utilized in treating CMF, including percutaneous cryoablation and radiofrequency ablation [15,16].

Our case series has a few clinical implications for the future management of CMF and can help guide future research surrounding the topic. Based on our findings, observation with serial imaging should be incorporated into treatment algorithms for CMF as a viable option for carefully selected patients, especially those who are asymptomatic with lesions in surgically challenging locations. Future research should elucidate the radiographic and clinical indications that should prompt eventual operative intervention.

This study has several limitations. First, the small sample size of eight patients reflects the rarity of CMF but limits statistical analysis and generalizability. Second, the retrospective design introduces potential selection bias. Third, the relatively short follow-up for one conservatively managed patient (eight months) limits conclusions about long-term stability, though the other case with a 104-month follow-up provides reassurance. Fourth, the lack of standardized outcome measures, including quality of life assessments and functional scoring systems, limits our ability to comprehensively compare treatment modalities. Despite these limitations, this case series represents, to the best of our knowledge, the first and longest reported follow-up of conservatively managed CMF.

## Conclusions

Chondromyxoid fibroma is a rare, benign, locally aggressive tumor that does not have strong recommendations guiding its nonoperative or operative management. This retrospective case series illustrates a variety of treatment options, including observation with serial imaging, intralesional curettage with electrocautery or bony adjuvants, and en bloc resection. We found no recurrence or progression of CMF despite the treatment modality chosen. Importantly, this series provides, to our knowledge, the first evidence that observation with serial imaging may be a safe and effective strategy in carefully selected asymptomatic patients, especially when lesions are located in anatomically challenging sites. Our results add to the limited literature on CMF and suggest that observation with serial imaging may be considered in carefully selected cases. However, given the small sample size and retrospective design, these findings require validation through larger multi-institutional studies to establish standardized selection criteria for conservative management and to optimize surveillance protocols.
